# Transcriptome Analyses of Mosaic (MSC) Mitochondrial Mutants of Cucumber in a Highly Inbred Nuclear Background

**DOI:** 10.1534/g3.117.300321

**Published:** 2018-01-12

**Authors:** Tomasz L. Mróz, Sebastian Eves-van den Akker, Agata Bernat, Agnieszka Skarzyńska, Leszek Pryszcz, Madeline Olberg, Michael J. Havey, Grzegorz Bartoszewski

**Affiliations:** * Department of Plant Genetics Breeding and Biotechnology, Faculty of Horticulture Biotechnology and Landscape Architecture, Warsaw University of Life Sciences, 02-776, Poland; † Biological Chemistry, John Innes Centre, NR4 7UH Norwich, United Kingdom; ‡ School of Life Sciences, University of Dundee, DD1 5EH, United Kingdom; § International Institute of Molecular and Cell Biology, 02-109 Warsaw, Poland; ** Department of Horticulture, University of Wisconsin-Madison, 53706 Wisconsin; †† United States Department of Agriculture-Agricultural Research Service, University of Wisconsin-Madison, 53706 Wisconsin

**Keywords:** *Cucumis sativus*, mitochondrial mutant, nuclear–mitochondrial interaction, plant mitochondria, RNA-seq

## Abstract

Cucumber (*Cucumis sativus* L.) has a large, paternally transmitted mitochondrial genome. Cucumber plants regenerated from cell cultures occasionally show paternally transmitted mosaic (MSC) phenotypes, characterized by slower growth, chlorotic patterns on the leaves and fruit, lower fertility, and rearrangements in their mitochondrial DNAs (mtDNAs). MSC lines 3, 12, and 16 originated from different cell cultures all established using the highly inbred, wild-type line B. These MSC lines possess different rearrangements and under-represented regions in their mtDNAs. We completed RNA-seq on normalized and non-normalized cDNA libraries from MSC3, MSC12, and MSC16 to study their nuclear gene-expression profiles relative to inbred B. Results from both libraries indicated that gene expression in MSC12 and MSC16 were more similar to each other than MSC3. Forty-one differentially expressed genes (DEGs) were upregulated and one downregulated in the MSC lines relative to B. Gene functional classifications revealed that more than half of these DEGs are associated with stress-response pathways. Consistent with this observation, we detected elevated levels of hydrogen peroxide throughout leaf tissue in all MSC lines compared to wild-type line B. These results demonstrate that independently produced MSC lines with different mitochondrial polymorphisms show unique and shared nuclear responses. This study revealed genes associated with stress response that could become selection targets to develop cucumber cultivars with increased stress tolerance, and further support of cucumber as a model plant to study nuclear-mitochondrial interactions.

Plant mitochondrial DNAs (mtDNAs) are characterized by relatively large sizes compared to animals and fungi, varying from 66 kb in *Viscum scurruloideum* (Skippington *et al.* 2015) to over 11,300 kb in *Silene conica* (Sloan *et al.* 2012). Within the Cucurbitaceae family, sevenfold differences in mtDNA sizes exist, from 379 kb in watermelon (Alverson *et al.* 2010) to 2740 kb in melon (Rodríguez-Moreno *et al.* 2011). These large mtDNA size differences are due in part to the accumulation of repetitive sequences that can undergo recombination to produce subgenomic molecules (sublimons) of varying structures (Sloan *et al.* 2012). Relative frequencies of sublimons can change over time or generations—a process referred to as substoichometric shifting (Shedge *et al.* 2007; Wu *et al.* 2015)—and are associated with gene-expression differences and increased tolerance to abiotic stresses (Shedge *et al.* 2010).

Mitochondrial-nuclear interactions are important for plant performance (Kihira 1982; Shen *et al.* 2015), and mitochondrial function can be affected by stress (Millar *et al.* 2003; Vanlerberghe *et al.* 2009; Gill and Tuteja 2010; Juszczuk *et al.* 2011; Atkin and Macherel 2009; Mróz *et al.* 2015), mutations or rearrangements in the mtDNA (Lauer *et al.* 1990; Newton *et al.* 1990; Hunt and Newton 1991; Gu *et al.* 1993; Hartmann *et al.* 1994; Marienfeld and Newton 1994; Sakamoto *et al.* 1996; Janska *et al.* 1998; Lilly *et al.* 2001; Kuzmin *et al.* 2005; Juszczuk *et al.* 2007), or substochiometric shifting (Shedge *et al.* 2007). Mitochondrial mutations are often deleterious and show chlorosis of leaves, slower growth, and reduced fertility as exemplified by the NCS mutations of maize (Coe 1983; Newton and Coe 1986; Newton 1995), *chm*-conditioned mutants of *Arabidopsis thaliana* (Martínez-Zapater *et al.* 1992; Abdelnoor *et al.* 2003), and the mosaic (MSC) mutants of cucumber (Malepszy *et al.* 1996; Lilly *et al.* 2001; Bartoszewski *et al.* 2007). Beneficial mitochondrial mutants also exist, such as systems of cytoplasmic male sterility (CMS) used to produce hybrid seed of many crop plants (Havey 2004).

Cucumber is a useful plant for mitochondrial genetics because, at 1.6 Mb, its mtDNA is among the largest known (Alverson *et al.* 2011) and shows paternal transmission (Havey 1997). MSC mitochondrial mutants of cucumber have been isolated after passage of the highly inbred, wild-type line B through cell-culture systems such as protoplast regeneration, cell suspension regeneration, or callus culture (Malepszy *et al.* 1996; Ładyżyński *et al.* 2002). Regenerated plants or progenies show paternal transmission of leaf mosaicism, slower growth, smaller aberrant flowers and fruits, and smaller seed with relatively poor germination (Malepszy *et al.* 1996). MSC lines originating from different cell cultures are phenotypically distinct (Ładyżyński *et al.* 2002; Bartoszewski *et al.* 2007), and possess complex rearrangements in their mtDNAs, including large deletions, duplications, and under-represented regions (Lilly *et al.* 2001; Bartoszewski *et al.* 2004; Del Valle-Echevarria *et al.* 2015). For example, MSC3 was derived from liquid meristematic culture and possesses an under-representation of the mitochondrial polycistronic region carrying *nad5ex4-atp4-nad5ex5*; MSC12 was regenerated from salt-resistant callus and MSC16 after leaf-callus culture, and both lines carry significantly fewer copies of the mitochondrial *rps7* coding region as well as different genomic deletions that carry no obvious coding regions (Del Valle-Echevarria *et al.* 2015). Biochemical analyses of MSC16 showed that mitochondrial complex I and chloroplast metabolism are negatively affected (Juszczuk *et al.* 2007; Szal *et al.* 2008, 2009). In this study, we used RNA-seq to compare among the transcriptomes of independently derived MSC lines relative to each other and their wild-type progenitor inbred B. We were interested to identify shared nuclear responses that could become targets of selection or gene editing to develop plants more tolerant to environmental stresses.

## Materials and Methods

### Plant materials

The highly inbred (>S_18_) wild-type line B of cucumber originated from the Polish cultivar “Borszczagowski” and was selected for regeneration ability from cell cultures (Malepszy *et al.* 1996). MSC lines 3, 12, and 16 were derived independently from different cultures all established using line B (Malepszy *et al.* 1996; Bartoszewski *et al.* 2004). Plants regenerated from cell cultures were selected for MSC phenotype in R_0_ (MSC3) or R_1_ (MSC12 and MSC16) generations and subsequently self-pollinated (>S_6_) to develop lines with stable expression of MSC (Bartoszewski *et al.* 2007). Line B, is monoecious producing both male and female flowers, and plants were self-pollinated using female and male flowers from the same plant.

**Figure 1:**
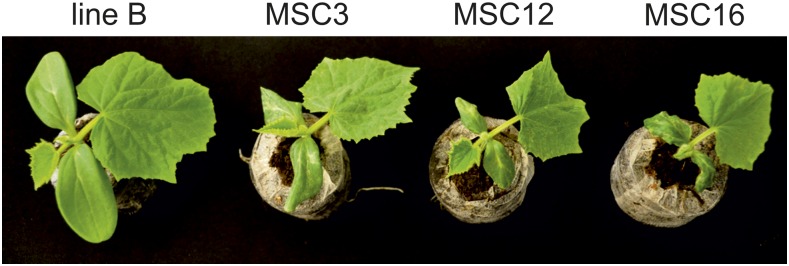
Representative developmental stage of plants of wild-type inbred B and the three MSC mitochondrial mutants sampled for transcriptome sequencing and validation of gene-expression differences.

Seeds were surface-sterilized in a 10% (v/v) solution of commercial bleach (final concentration of 0.45% sodium hypochlorite) for 5 min, washed three times in distilled water and placed on wet filter paper to imbibe for 24 hr at 28°. Imbibed seeds were sown into peat pots Jiffy-7 (Jiffy International AS, Kristiansand, Norway) at pH 6.0, and grown in phytotron SANYO MLR-350H (SANYO, Osaka, Japan). For each line, 12 plants were grown at 25/20° day/night and a 16-hr photoperiod under fluorescent white light 400 μmol m^−1^ s^−2^ and a relative humidity of 55–65%. Because MSC12 and 16 are slower growing, seeds were planted 2 d earlier than MSC3 and inbred B. Upper plant parts were harvested after cutting 0.5 cm below the cotyledons when plants possessed fully expanded first true leaves at ∼12 d after sowing for line B and MSC3 and 14 d for MSC12 and MSC16 (Figure 1). Tissues from six plants of each line were pooled and immediately frozen in liquid nitrogen. Two biological replications were used for sequencing, and three for validations using real-time quantitative PCR (RT-qPCR).

### RNA extraction

Total RNA was isolated using Concert Plant RNA Reagent (Invitrogen, Carlsbad, CA). RNA was treated with DNAse I (TURBO DNA-free kit; Ambion Inc., Austin, TX) and PCR checked for lack of DNA contamination using specific primers to actin CsActF37 and CsActR438 (Mizuno *et al.* 2002). The concentrations and the preliminary qualities of RNAs (260/280 and 260/230 ratios) were measured with a NanoDrop 2000 Spectrophotometer (Thermo Fisher Scientific, Waltham, MA) before and after DNase I treatment. RNA integrity was checked by measuring RNA Quality Index (RQI) using Experion Automated Electrophoresis System according to manufacturer’s instructions (Bio-Rad Laboratories, Hercules, CA). Samples of RNA used for sequencing had total amounts of RNA ≥ 22 µg, concentrations ≥ 400 ng/µl, major ribosomal subunit ratio 28S:18S ≥ 1, and RQI ≥ 8.

### Construction and sequencing of normalized and non-normalized cDNA libraries

The four normalized cDNA libraries (one each from line B and MSC lines 3, 12, and 16) were synthesized from the total RNAs using the SMART approach (Zhu *et al.* 2001) by Evrogen (Moscow, Russia). Normalization used the duplex-specific nuclease (DSN) method (Zhulidov *et al.* 2004), which included cDNA denaturation and reassociation, treatment by DSN (Shagin *et al.* 2002), and amplification of the normalized fraction by PCR using SMART primers. The four normalized libraries were sequenced as single reads using the Illumina HiSeq2000 platform as recommended by the manufacturer (Illumina, San Diego, CA).

Using total RNAs from two biological replications of line B and the three MSC lines, eight non-normalized libraries were synthesized at BGI-Tech (Shenzhen, China). RNA integrity was confirmed using Bioanalyzer 2100 (Agilent Technologies, Santa Clara, CA). Polyadenylated messenger (m) RNA was purified using oligo(dT) magnetic beads. mRNA was sheared to fragments of ∼200 bp and used as template for cDNA synthesis with random hexamers. Sequencing adapters were ligated after end repair of cDNAs. Gel electrophoresis was used for cDNA purification and suitable fragments were enriched by PCR. Libraries were multiplexed and paired-end sequenced in a single run using the Illumina HiSeq2000 (Illumina). Sequence reads from the normalized and non-normalized libraries were deposited in National Center for Biotechnology Information (NCBI, Bethesda, MD).

### Differential gene expression analysis and gene ontology (GO) annotation

For both normalized and non-normalized libraries, reads were aligned to the nuclear reference genome of Chinese Long 9930 v2 cucumber (Huang *et al.* 2009). A reference index for normalized libraries was created using the Bowtie-build function (Bowtie2 v2.2.3; Langmead and Salzberg 2012), and each read set was aligned to the reference using TopHat2 (v2.0.12; Kim *et al.* 2013) with default settings. Read counts mapping to genes were determined using Cuffdiff tool of the Cufflinks program (Trapnell *et al.* 2012) and provided estimates of the fragments per kilobase of transcript per million mapped reads (FPKM) for each transcript. Differential expression was determined as fold-change of FPKM for each MSC mutant (FPKM_MSC) compared to B (FPKM_B) calculated as log2(FPKM_MSC/FPKM_B), and values were used to cluster lines and gene expression using Gene Cluster version 3.0 (de Hoon *et al.* 2004). Centroid linkage and hierarchical clustering were completed using Pearson’s uncentered correlation, and visualized using TreeView (Saldanha 2004). Given the large number of differentially expressed genes (DEGs), Gene Cluster was then used to filter data to only include genes that had expression differences of at least twofold compared to B, such that |log2(FPKMMSC/FPKMB)| ≥ 1.0 for at least one of the MSC lines, and hierarchical clustering and visualization were completed as before. Sets of uniquely and differentially transcribed genes were identified for each MSC line by selecting clusters of expression profile similar among mutants, within MSC12 and MSC16, and unique to MSC3. The GO enrichment tool at the cucurbit genome database (http://www.icugi.org/) was used to determine ontology of DEG clusters. Uncorrected *p*-values from tail-end hypergeometric distribution where *p* ≤ 0.05 were considered significant and GO terms with at least 4% cluster frequency were selected.

For non-normalized libraries, raw sequencing reads were processed using Trimmomatic (v0.32; Bolger *et al.* 2014) to remove over-represented sequences and low-quality bases using a minimum phred quality score of 22 and minimum length of read of 50 nt. High-quality paired-end reads (after trimming) were mapped to the reference of 9930 v2 (Huang *et al.* 2009) using TopHat2 (v2.0.13; Kim *et al.* 2013). Sorting and indexing of mapping results was performed using the SAMtools package (v0.1.19; Li *et al.* 2009). Read counts for each gene were determined using Bedtools (v2.16.2; Quinlan and Hall 2010) and then normalized [trimmed mean of M values (TMM)] and calculated using Trinity wrapper scripts (r20131110; Haas *et al.* 2013) for EdgeR Bioconductor (Robinson *et al.* 2010) to identify DEGs in the MSC mutants relative to wild-type line B. DEGs were identified using a fold change ≥4 and *p*-value <0.01, and were clustered based on their expression profile at 40% tree height. Sequences of DEGs were blasted against NCBI protein database using default settings. The Blast2GO (v2.7.2, Conesa *et al.* 2005) was used to retrieve GO terms and annotations using all available databases in InterProScan. Functional annotations were enriched by alignment to the TAIR10 proteins database (http://www.arabidopsis.org/) and identification of the *A. thaliana* orthologs.

### cDNA synthesis and RT-qPCR

Concentrations of high-quality RNAs were adjusted to 300 ng/µl and cDNAs synthesized using the Transcriptor High Fidelity cDNA Synthesis Kit (Roche, Basel, Switzerland) in 20 μl reactions using oligo(dT) primers according to manufacturer’s instructions. RT-qPCR analyses were performed using diluted cDNA (1:11.5) reverse transcribed from 2.1 μg of total RNA, and carried out using CFX96 Touch cycler (Bio-Rad Laboratories) with Master Mix Maxima SybrGreen qPCR MM 2× ROX (Thermo Fisher Scientific), according to the manufacturer’s instructions, using 4 μl of cDNA in each reaction. The PCR program was 50° for 2 min pretreatment, initial denaturation at 95° for 10 min, followed by 40 cycles of 15 sec at 95° and 1 min at 58°. Melting curve analysis was performed immediately after RT-qPCR. The temperature range used for the melting curve generation was from 70 to 95° (increments of 0.5°). Parameters of RT-qPCR were established by series of preliminary experiments with various PCR cycle number and annealing temperature gradient (55–65°). The mean efficiency of amplification in RT-qPCR was assessed using LinRegPCR (v2013.1; Ramakers *et al.* 2003) based on linear regression calculated for the slope of the regression line in the exponential growth phase of the product for each sample individually.

### Validation of expression profiles of DEGs

Eleven candidate genes were evaluated as potential references for RT-qPCR (Supplemental Material, Table S1 in File S1), and 24 independent reactions (three biological × two technical replications for each of the four cucumber lines) were performed for each candidate gene as described previously. Results were analyzed using three algorithms: geNorm (v3.4; Vandesompele *et al.* 2002), NormFinder (v0.953; Andersen *et al.* 2004), and BestKeeper (v1; Pfaffl *et al.* 2004). The final ranking was established by calculating the geometric mean (GeoMean) for the data generated by each applet based on the method developed by Żyżyńska-Granica and Koziak (2012). Genes *TIP41*, *F-box* and *UBI-ep* showed the lowest geometric mean (GeoMean) and greatest stability of expression (Table S2 in File S1), and were chosen as references for RT-qPCR.

Primers for all DEGs from the non-normalized libraries were designed using the CDS sequences (Huang *et al.* 2009; http://www.icugi.org/) and Primer3 (Koressaar and Remm 2007; Untergrasser *et al.* 2012) (Table S3 in File S1). Initially, the primers were tested on mixed, equal amounts of cDNAs from all lines by RT-qPCR as described above using three biological and three technical replicates, and four negative controls. Relative normalized expression (2^−ΔΔCt^ method) of DEGs and statistical analysis (Student’s *t*-test) were performed to determine significant differences using software CFX Manager (v3.1; Bio-Rad Laboratories). Expression-level differences between the *msh1* mutant and wild-type *Arabidopsis* were evaluated for the validated DEGs between MSC lines and inbred B using microarray data from NCBI accession PRJNA151951.

### Detection of hydrogen peroxide (H_2_O_2_)

H_2_O_2_ accumulation is commonly used as a stress indicator in plants (Inzé *et al.* 2012), and amounts were estimated by visualization after infiltration of leaves with 3,3′-diaminobenzidine (DAB) (Thordal-Christensen *et al.* 1997). Polymerization of DAB with H_2_O_2_ in the presence of peroxidase produces a brown product, which was detected using a Leica M165FC stereo microscope (Leica Microsystems, Wetzlar, Germany) as described by Wituszyńska *et al.* (2015). Detection was performed on leaves of six independent plants of each line grown under optimal conditions, including wild-type B. The positive control was six leaves of inbred B that had been repeatedly squeezed using tweezers to produce mechanical stress points.

### Data availability

Sequence data from this study are available at NCBI GenBank as BioProject PRJNA382994 and Sequence Read Archives (SRA) SAMN06768506–SAMN06768517.

## Results

### Sequencing of normalized cDNA libraries

**Figure 2:**
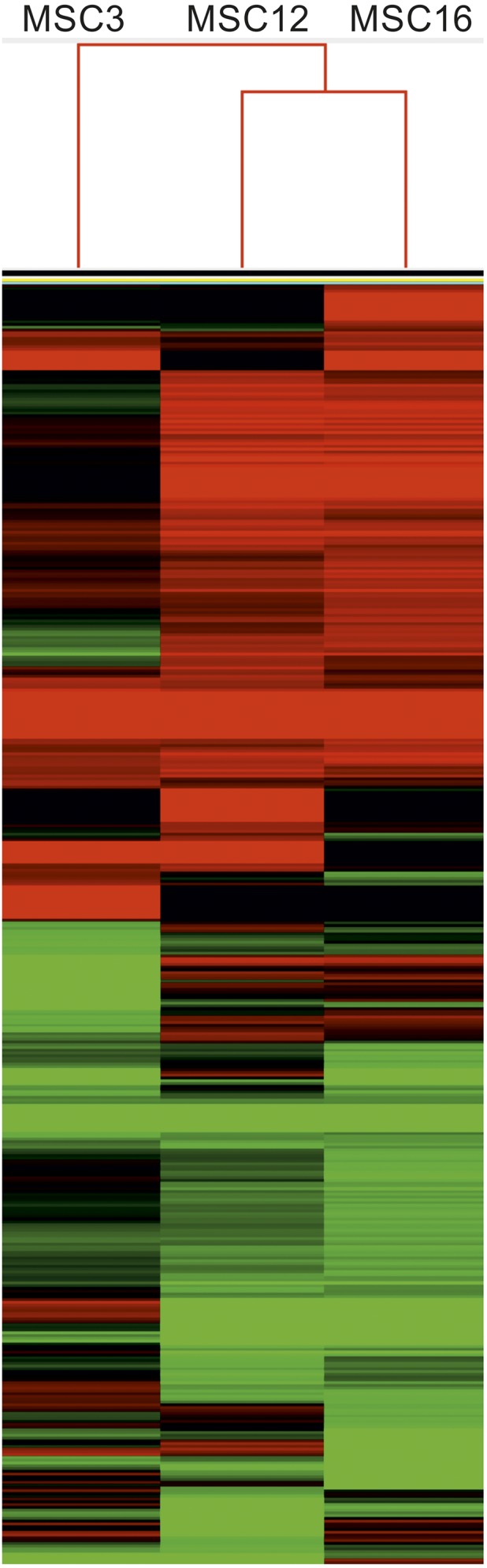
Clustering of genes from normalized cDNA libraries showing at least twofold expression difference in mitochondrial mutants MSC3, 12, and 16 relative to wild-type inbred B. Red lines indicate upregulation and green downregulation of genes. A hierarchical clustering was completed using Pearson’s uncentered correlation and visualized using the TreeView software.

We initially sequenced normalized cDNA libraries from MSC lines 3, 12, and 16, and their wild-type progenitor B to determine if independently produced MSC lines show evidence of unique gene-expression profiles relative to each other and B. Total numbers of reads were between 50.6 and 59.3 million for each line, of which 50, 56, 42, and 49% mapped to the 9930 v2 reference for inbred B and MSC 3, 12, and 16, respectively; >1000 genes showed at least twofold differential expression for each MSC line compared to B. For MSC12 and MSC16, clusters of 1714 genes were upregulated and 1476 genes downregulated relative to B, and uniquely expressed compared to MSC3. MSC3 had clusters of 281 genes that were uniquely upregulated and 699 genes uniquely downregulated compared to inbred B and the other two MSC mutants. Clustering of gene expression patterns clearly showed more similarity between MSC12 and MSC16 *vs.* MSC3 (Figure 2). The similarity of gene expression shared by MSC12 and MSC16 relative to MSC3 provides evidence for specific nuclear responses to mitochondrial mutations in the same highly inbred nuclear background. GO annotations of gene clusters that were upregulated or downregulated in all three MSC lines compared to inbred line B were generally associated with catalytic activity, transcription factors, and oxidoreductase activity. These differences are likely the result of stress responses due to dysfunctional mitochondria. Previous studies have shown altered oxidation-reduction and accumulation of reactive oxygen species (ROS) in MSC16 relative to B (Szal *et al.* 2009; Juszczuk *et al.* 2011).

### Sequencing of non-normalized cDNA libraries

**Figure 3:**
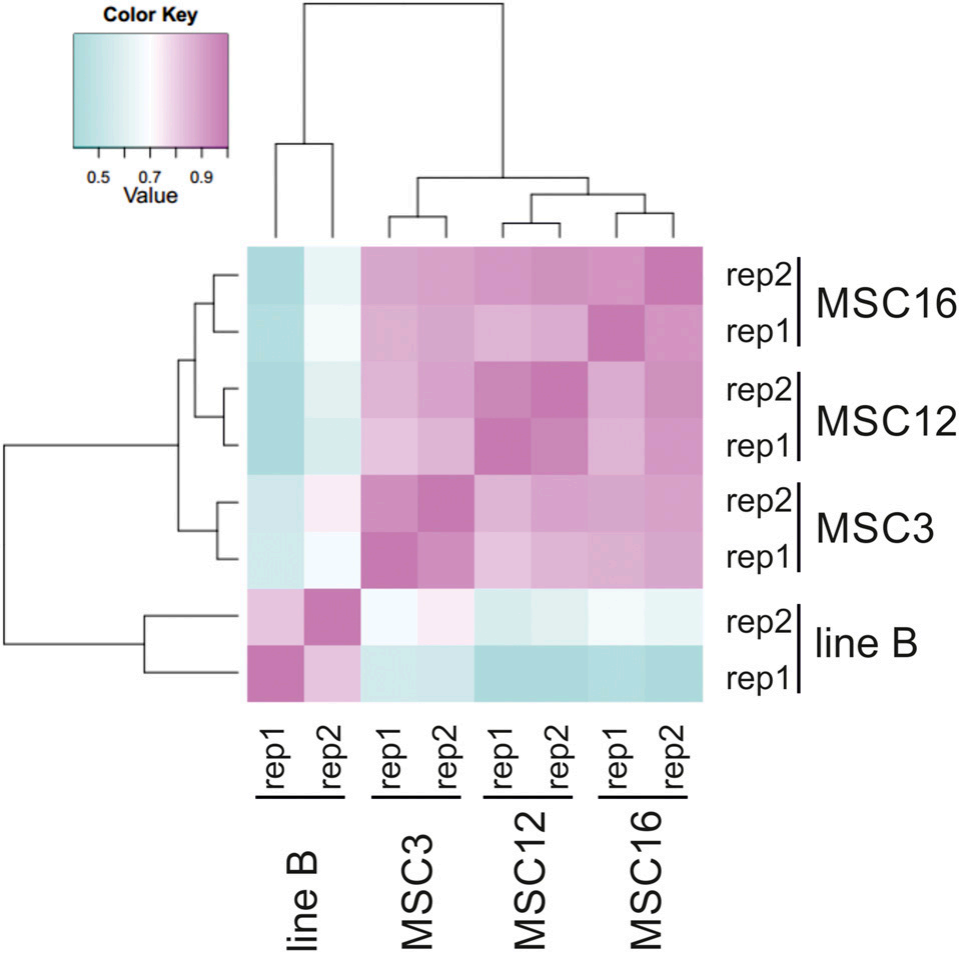
Hierarchically clustered Spearman correlation matrix resulting from pairwise comparison of transcript expression values (TMM-normalized FPKM) for each pair of samples for mitochondrial mutants MSC3, 12, and 16 and wild-type inbred B for the first (rep1) and second (rep2) biological replications. The results were visualized using Trinity wrapper scripts (r20131110) for EdgeR Bioconductor.

In order to identify and validate DEGs specific to all three MSC mutants compared to their progenitor inbred B, RNA-seq was performed on non-normalized libraries from two biological replications of each line. Sequencing yielded >25 million raw reads from both replicates for each line. After quality checking, reads were trimmed and mapped to the cucumber 9930 v2 reference. Numbers of mapped reads were between 15.9 and 16.9 million for each line, resulting in an average read-mapping rate of 83% (Table S4 in File S1). We identified 41 DEGs with minimum fourfold differences shared by all three MSC mutants relative to wild-type B, and 40 of these DEGs were upregulated and one downregulated (Table 1). Pairwise comparisons of DEGs expression profiles between line B and each MSC mutant were carried out using the Spearman’s rank correlation coefficient, and revealed the highest correlation between inbred B and MSC3 (Figure 3). Expression profiles of MSC12 and 16 were correlated, and both mutants clustered into a separate subgroup (Figure 3).

**Table 1:** Functional annotation of 41 DEGs shared by three MSC mitochondrial mutants relative to wild-type cucumber

No.	Cucumber unigene	Abbr.	Functional Annotation		*A. thaliana* ortholog
			Blast2GO	TAIR10 *A. thaliana*	
**DNA REPAIR MECHANISMS**					
1	Csa3M819830	WRNexo	werner syndrome-like exonuclease-like	protein with homology to the exonuclease domain of hWRN-p of human protein Werner Syndrome Exonuclease (WEX)	AT4G13870
2	Csa5M577370	NUDIX1*^a^*^*2*^	nudix hydrolase 1-like	NUDIX hydrolase homolog 1	AT1G68760
**REGULATION OF TRANSCRIPTION**					
3	Csa2M193320	BHLH92	transcription factor bhlh92	BHLH92; basic helix-loop-helix (bHLH) DNA-binding superfamily protein	AT5G43650
4	Csa4M193250	NAC87	nac domain-containing protein 100-like	NAC domain containing protein 87 (ANAC087)	AT5G18270
5	Csa6M042450	CRF6	ethylene-responsive transcription factor crf6-like	cytokinin response factor 6	AT3G61630
6	Csa6M518170	NAC73	nac domain-containing protein 21	NAC domain containing protein 73 (NAC073)	AT4G28500
7	Csa7M170600	RL1	transcription factor radialis-like	ATRL1, RAD-LIKE 1, RADIALIS-LIKE SANT/MYB 2, RL1, RSM2	AT4G39250
**SIGNAL TRANDUCTION MECHANISMS**					
8	Csa3M167380	CaBP1	Ca^2+^-binding protein 1	Ca^2+^ binding protein 1 (CP1)	AT5G49480
**CELLULAR METABOLIC PROCESSES**					
9	Csa1M703040	nd*^b^*	uncharacterized atp-dependent helicase-like isoform 2	P-loop containing nucleoside triphosphate hydrolases superfamily protein	AT1G65810
10	Csa4M303690	GSTu8*^a^*^*2*^	glutathione s-transferase u8-like	glutathione S-transferase TAU 8	AT3G09270
11	Csa4M304250	GST-like*^a^*^*2*^	probable glutathione s-transferase-like	glutathione S-transferase TAU 7	AT2G29420
12	Csa4M639960	unAT*^a^*^*2*^	uncharacterized acetyltransferase at3g50280-like	HXXXD-type acyl-transferase family protein	AT5G42830
13	Csa1M537480	1-BBE	reticuline oxidase-like	FAD-binding Berberine family protein	AT1G30700
14	Csa1M539350	2-BBE	reticuline oxidase-like	FAD-binding Berberine family protein	AT5G44400
15	Csa1M595860	CYP-like*^a^*^*1*^	secologanin synthase-like	putative cytochrome P450 (CYP72A15)	AT3G14690
16	Csa4M285790	POX53	peroxidase 53-like	peroxidase 53, PRX53	AT5G06720
17	Csa6M094680	PPI1	proton pump-interactor 1-like	proton pump interactor 1	AT4G27500
18	Csa6M094690	PPI2	proton pump-interactor 2-like isoform x1	uncharacterized protein with putative role in response to salt stress	AT1G10880
19	Csa6M517010	NDA2	internal alternative nad h-ubiquinone oxidoreductase mitochondrial-like	alternative NAD(P)H dehydrogenase 2 (NDA2)	AT2G29990
20	Csa6M517020	NDA1	internal alternative nad h-ubiquinone oxidoreductase mitochondrial-like	internal NAD(P)H dehydrogenase in mitochondria (NDA1)	AT1G07180
**RESPONSE TO STIMULUS**					
21	Csa3M020080	1-HSP23	small heat shock chloroplastic-like	mitochondrion-localized HSP23.6	AT4G25200
22	Csa3M020090	2-HSP23	small heat shock chloroplastic-like	mitochondrion-localized HSP23.6	AT4G25200
23	Csa3M829160	SAP12	zinc finger an1 domain-containing stress-associated protein 12-like	putative zinc finger protein (PMZ), SAP12, Stress-associated protein 12	AT3G28210
**REGULATION OF PROTEOLYTIC PROCESSES**					
24	Csa2M360680	1-SerpinZX	serpin-ZX-like	serine protease inhibitor (SERPIN) family protein	AT1G47710
25	Csa2M360690	2-SerpinZX	af284038_1phloem serpin-1		
26	Csa5M590010	Ntn	N-terminal nucleophile aminohydrolases (ntn hydrolases) superfamily protein isoform 1	N-terminal nucleophile aminohydrolases (Ntn hydrolases) superfamily protein	AT3G26340
27	Csa6M504470	1-unFtsH	AAA-ATPase At2g18193-like	P-loop containing nucleoside triphosphate hydrolases superfamily protein	AT2G18193
28	Csa6M504480	2-unFtsH	AAA-ATPase At2g18193-like	P-loop containing nucleoside triphosphate hydrolases superfamily protein	AT2G18193
**TRANSPORT OF METAL IONS/LIPIDS**					
29	Csa1M476010	HIPP26	heavy metal-associated isoprenylated plant protein 26-like	heavy metal transport/detoxification superfamily protein	AT1G06330
30	Csa4M141240	1-nsLTP2*^a^*^*2*^	non-specific lipid-transfer protein 2-like	bifunctional inhibitor/lipid-transfer protein/seed storage 2S albumin superfamily protein;	AT1G48750
31	Csa4M146250	2-nsLTP2*^a^*^*2*^	non-specific lipid-transfer protein 2-like	bifunctional inhibitor/lipid-transfer protein/seed storage 2S albumin superfamily protein;	AT1G48750
**OTHERS**					
32	Csa1M600240	BPM4	btb poz and math domain-containing protein 4-like isoform 3	nd*^b^*	nd*^b^*
33	Csa2M351860	nd*^b^*	unknown protein	unknown protein	nd*^b^*
34	Csa3M120450	nd*^b^*	unknown protein	unknown protein	nd*^b^*
35	Csa3M778180	nd*^b^*	unknown protein	unknown protein	nd*^b^*
36	Csa4M639110	nd*^b^*	unknown protein	unknown protein	nd*^b^*
37	Csa5M155520	HAP2	protein hapless 2-like	HAPLESS 2/Generative cell-specific 1 is a gamete fusion protein	AT4G11720
38	Csa5M606890	nd*^b^*	unknown protein	unknown protein	nd*^b^*
39	Csa6M006690	unPLAC	survival motor neuron isoform x4	PLAC8 family protein	AT5G41390
40	Csa6M154530	PGA55	probable gpi-anchored adhesin-like protein pga55	protein of unknown function (DUF688)	AT2G30990
41	Csa7M304870	nd*^b^*	unknown protein	unknown protein	nd*^b^*

a Orthologs differentially regulated in the *MutS Homolog1* (*msh1*) mutant vs. control of *A. thaliana* with transcription higher in *msh1* (^*1*^) or wild-type (^*2*^) control (Shedge et al. 2007).

b Not determined.

**Figure 4:**
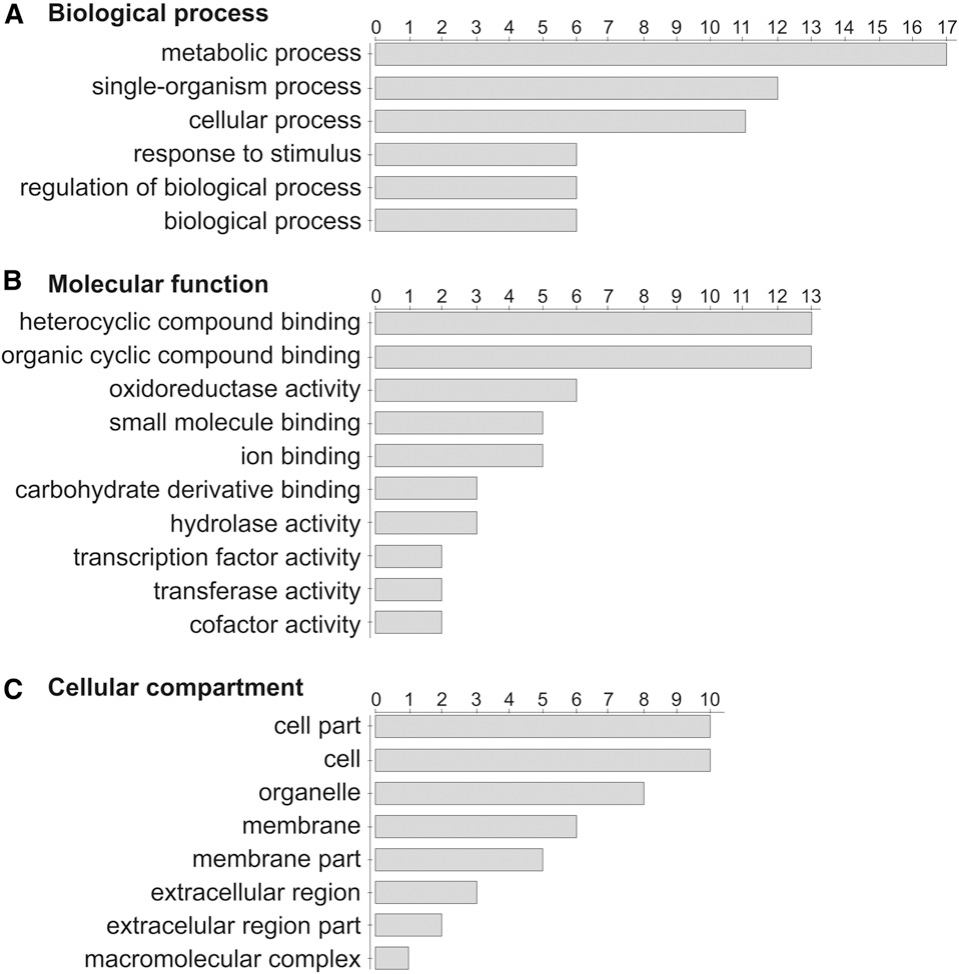
GO of DEGs shared by three MSC mitochondrial mutants compared to wild-type line B used to annotate putative (A) biological process, (B) molecular function, and (C) cellular compartment. The number of genes for each category is shown. The results were calculated and visualized using Blast2GO v2.7.2.

Of the 41 DEGs, six showed no similarity to the cucumber 9930 v2 reference and TAIR10 *A. thaliana* protein database using Blast2GO. For the remaining 35 DEGs, GO classifications were used to identify putative cellular locations, and biological and molecular functions (Figure 4), and were assigned to eight groups: DNA repair mechanism, regulation of transcription, signal transduction, cellular metabolic processes, response to stimulus, regulation of proteolytic processes, transport of metal ions/lipids, and other proteins (Figure 4 and Table 1). The largest group possessed 12 genes encoding proteins involved in cellular metabolic processes (including oxidoreductase activity), and five genes for each of regulation of transcription and proteolytic processes (Table 1); 23 of the DEGs were directly or indirectly associated with stress-response pathways (Table S5 in File S1).

### DEGs shared by all MSC lines

**Figure 5:**
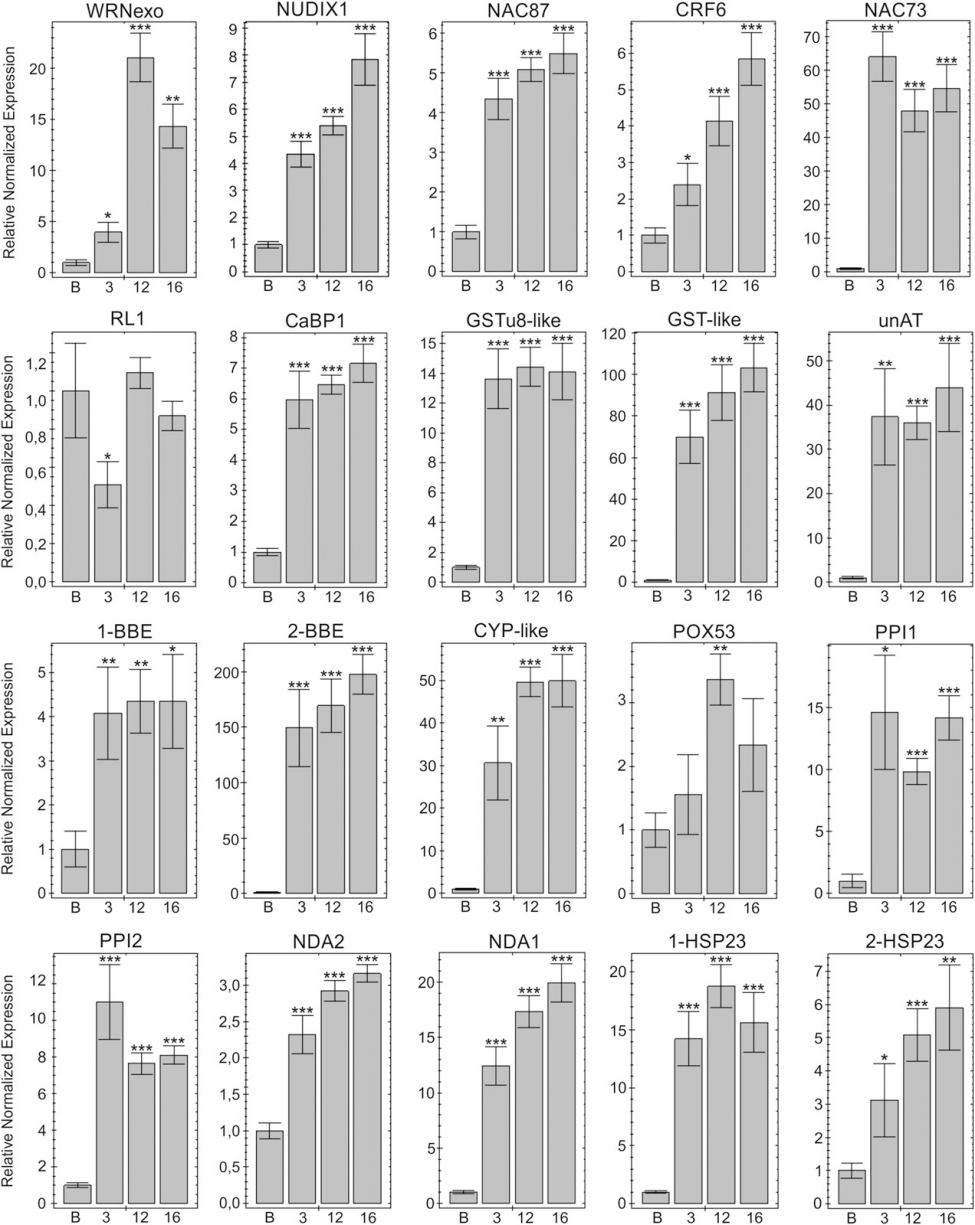

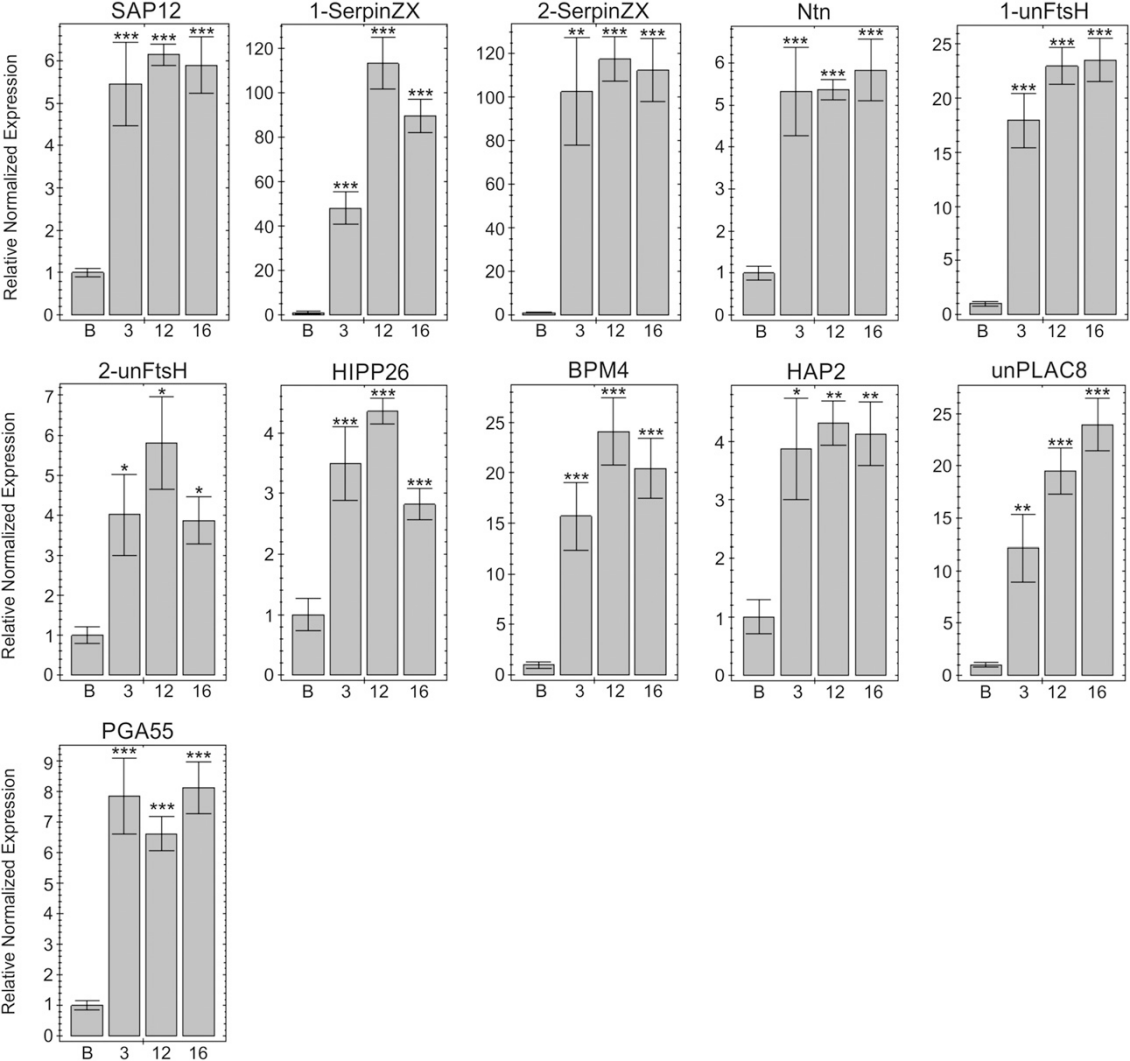
Validated differential gene expression between wild-type inbred B and mitochondrial mutants MSC3, 12, and 16. Diagrams show the average relative gene expression levels normalized to line B (assigned value of 1) ± SEM. Gene names are described in Table 1. Significance levels are *p* < 0.05 (*), 0.01 (**), and 0.001 (***). Due to the wide range of differences in the expression between target genes, different scales of the Relative Normalized Expression were used (from 0–1.2 to 0–200). The results were calculated and visualized using software CFX Manager (v3.1; Bio-Rad Laboratories).

Thirty-one DEGs were selected for validation using RT-qPCR, of which 30 were upregulated and one gene (RL1) downregulated in the MSC lines compared to wild-type B (Figure 5 and Table S6 in File S1). The three DEGs with the highest expression level differences were reticulin oxidase-like protein (BBE-2 with a 172-fold change) and two serine protease inhibitors (1-SerpinZX and 2-SerpinZX with 84-fold and 111-fold changes, respectively). Differences in relative expression profiles between MSC lines were also observed (Figure 5 and Table S6 in File S1). Expression levels of nine genes were significantly higher in MSC12 and 16 compared to MSC3 (WRNexo, NUDIX1, CRF6, CYP-like, NDA1, NDA2, 1-SerpinZX, 1-unFtsH, and unPLAC8). Five DEGs showed significantly lower expression in MSC16 than MSC12 (WRNexo, 1-SerpinZX, 2-unFtsH, and HIPP26) or MSC3 (PPI2). Two genes showed different expression levels in only one mutant, RL1 in MSC3 and POX53 in MSC12.

### Elevated levels of H_2_O_2_ in MSC mutants

**Figure 6:**
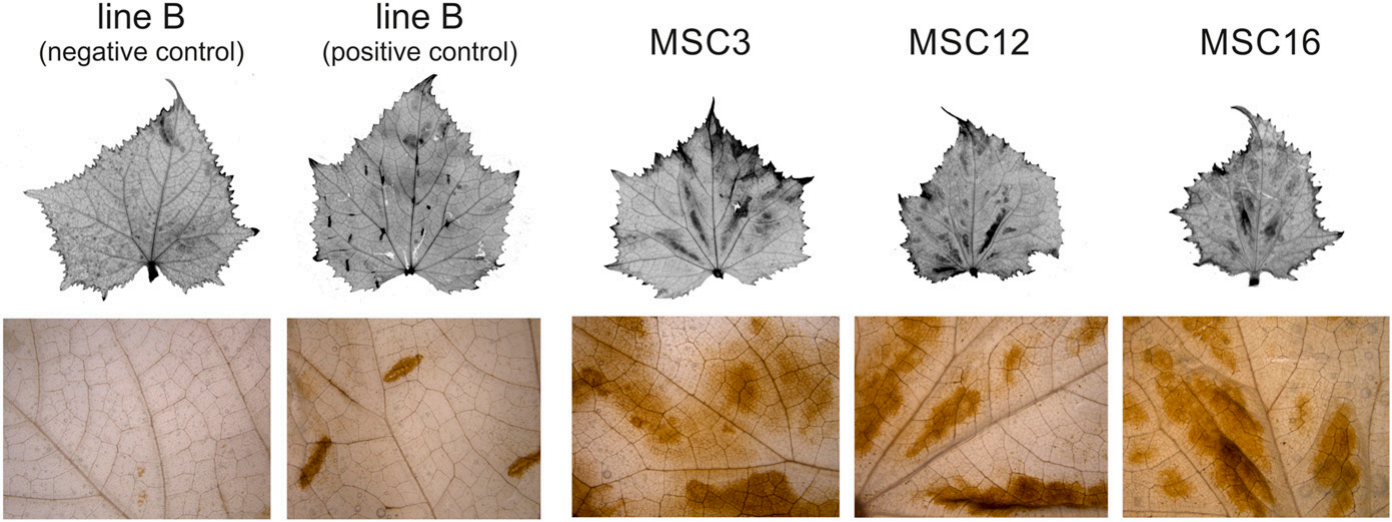
Images showing increased accumulation of hydrogen peroxide (H_2_O_2_) in leaves of MSC mitochondrial mutants and controls grown under optimal conditions. Negative control is wild-type inbred B; positive control is inbred B with points of mechanical stress induced by squeezing leaves with tweezers. The results were visualized using a Leica M165FC stereo microscope system (Leica Microsystems).

DAB staining revealed increased production of H_2_O_2_ in leaves of MSC mutants relative to wild-type B (Figure 6). The brown product of polymerization of DAB and H_2_O_2_ appeared as areas of irregular shapes between the vascular bundles in leaves. Higher amounts of H_2_O_2_ are consistent with upregulation of stress response genes in the MSC mutants relative to wild-type B.

## Discussion

Cucumber MSC mutants represent a unique system to produce mitochondrial mutants and study nuclear responses in a highly inbred nuclear background. Independently derived MSC lines were isolated after passage of the highly inbred, wild-type line B through cell cultures, and possess distinct phenotypes (Ładyżyński *et al.* 2002) and different rearrangements in their mtDNAs (Bartoszewski *et al.* 2007). The three MSC lines used in this study possess regions in their mtDNAs that are under-represented relative to wild-type B. MSC12 and 16 share an under-representation of the *rps7* coding region. MSC12 has larger regions of its mtDNA under-represented relative to MSC16 and B; however, these regions do not carry any obvious mitochondrial genes (Del Valle-Echevarria *et al.* 2015). MSC3 possesses an under-representation of the *nad5ex4-atp4-nad5ex5* polycistronic region (Del Valle-Echevarria *et al.* 2015). It cannot be ruled out that the MSC lines might harbor tissue-culture-induced changes in their nuclear genomes. However, it is very unlikely that the MSC phenotype would be caused by mutations at nuclear loci affecting the prevalence of mitochondrial genomes, because independent mutation would have to occur at least three times giving rise to three lines (MSC3, 12, and 16) with the same paternally transmitted phenotype (Malepszy *et al.* 1996; Lilly *et al.* 2001). Paternal imprinting of nuclear alleles as the genetic basis for the MSC phenotype was eliminated through a series of the reciprocal crosses, self-pollinations through the F3 generation, and multiple test-crosses of the MSC16 line (Lilly *et al.* 2001). These similarities and differences in the mtDNAs of independently derived mitochondrial mutants allow for comparison of their nuclear transcriptomes in the highly inbred nuclear background of wild-type line B. We expected that the MSC lines would differ from B for nuclear stress-response genes due to mitochondrial dysfunction (Juszczuk *et al.* 2007; Szal *et al.* 2009). Because MSC12 and MSC16 share an under-representation of the *rps7* coding region, we hypothesized that nuclear responses would be more similar between these two mutants relative to MSC3 and inbred B, and that MSC3 would show uniquely transcribed nuclear genes relative to MSC12 and 16. Clustering of nuclear transcripts from normalized libraries showing at least twofold expression differences in the MSC lines relative to B clearly revealed unique nuclear responses to the different mitochondrial mutations (Figure 2).

We sequenced from replicated cDNA libraries and bioinformatically identified 41 DEGs with at least fourfold changes in expression levels shared among three MSC lines relative to B, as well as transcriptional differences unique to the MSC lines. Expression differences for 31 DEGs were validated using RT-qPCR, of which 30 DEGs showed increased expression in the MSC lines compared to wild-type progenitor B, and one gene (RL1) was downregulated (Figure 5). Differences were observed between the initial bioinformatic analyses and subsequent validation by RT-qPCR, and two genes showed altered expression in only one MSC mutant (RL1 in MSC3 and POX53 in MSC12). Gene expression differences among the MSC mutants were also validated; nine genes had expression levels significantly higher in MSC12 and 16 than MSC3, and five genes showed expression levels significantly lower in MSC16 than MSC12 or MSC3 (Figure 5). These expression differences reveal unique nuclear responses to different mitochondrial mutations.

MtDNA structure can significantly influence phenotype and mitochondrial function (Bartoszewski *et al.* 2007; Arrieta-Montiel and Mackenzie 2011; Gualberto *et al.* 2014). Rearrangements in the mtDNA can be induced by DNA repair processes such as recombination activated by double-strand breaks (DSBs) (Davila *et al.* 2011). These mtDNA rearrangements may occur naturally during plant development (Yesodi *et al.* 1995) or may be associated with environmental and/or stress conditions (Davila *et al.* 2011). Although mitochondrial genes in the angiosperms are evolutionarily conserved and show relatively low mutation rates, structural rearrangements in the mtDNA can lead to mitochondrial dysfunction (Gu *et al.* 1993; Gualberto *et al.* 2014). Mitochondrial dysfunction is often associated with accumulation of ROS and expression changes for many mitochondrial and nuclear genes, including nuclear genes encoding mitochondrial proteins (NGEMPs), to maintain cell homeostasis (Inzé *et al.* 2012). The results of this study suggest that under-representation of mitochondrial coding regions in the cucumber MSC mutants cause physiological stress, as evidenced by elevated levels of H_2_O_2_ in the leaves of MSC mutants relative to wild-type B (Figure 6), and DEGs are involved in the antioxidative defense system (Table 1, Table S4 in File S1).

As mentioned above, the studied MSC lines are characterized by unique mitochondrial genome rearrangements (Bartoszewski *et al.* 2007). However, despite structural differences in their mtDNAs, all three lines share a clear mosaic phenotype (Malepszy *et al.* 1996; Bartoszewski *et al.* 2007). Del Valle-Echevarria *et al.* (2015) reported that the MSC mutants do not appear to differ in mitochondrial protein composition, despite differences in mitochondrial gene copy number and transcript abundance. Our findings revealed that there are at least 41 genes whose expression is associated with specific nuclear responses to mitochondrial mutations in the same highly inbred nuclear background (Table 1). Presumably, it may be related to respiratory chain complex I dysfunction (Juszczuk *et al.* 2011), and the activation of an alternative route of electron transfer in MSC lines (Mróz *et al.* 2015). Thus, it seems that altered respiratory chain function is the primary source of a stress signal, and the identified DEGs are probably related to the response to this signal.

Mutations in nuclear genes such as *msh1* or *rec*A revealed rearranged mtDNA of *A. thaliana* that significantly affected gene expression and increased stress tolerances (Shedge *et al.* 2010; Davila *et al.* 2011). Similarly, the MSC mutants of cucumber possess rearranged mtDNAs, and we compared DEGs in the MSC mutants to those associated with the *msh1* mutant of *A. thaliana* (Shedge *et al.* 2010). Only one of the 41 DEGs identified in this study shared altered expression in both *msh1 Arabidopsis* and the MSC lines: upregulation of a CYP-like gene. Six DEGs showing higher expression in the MSC lines also showed increased expression in wild-type relative to *msh1 Arabidopsis* (NUDIX1, GSTu8, GST-like, unAT, 1-nsLPT2, and 2-nsLTP2). The relatively few DEGs shared between mitochondrial mutants of *Arabidopsis* and cucumber indicate that gene-expression analyses of plants with different mitochondrial mutations should reveal common and unique nuclear responses. Cucumber is a useful plant for these analyses because it possesses a large mtDNA with pockets of repetitive DNAs that undergo recombination to produce structurally rearranged molecules (Lilly *et al.* 2001; Alverson *et al.* 2010). These rearranged mtDNAs may exist as sublimons in wild-type cucumber, and/or be induced during passage through cell cultures and regeneration of plants. We propose that highly inbred or doubled-haploid lines of cucumber be passed through cell cultures and regenerated plants and their progenies screened for paternally transmitted, variant phenotypes such as MSC. Sequencing of the mtDNAs of these plants should reveal specific under-represented regions associated with the variant phenotypes (Del Valle-Echevarria *et al.* 2015). RNA-seq on a collection of unique mitochondrial mutants should reveal common nuclear responses, as well as unique transcriptional differences toward a better understanding of mitochondrial-nuclear interactions. Common nuclear responses may reveal stress response pathways that can be targets of selection or gene editing to develop plants showing greater tolerances to environmental stresses. For example, NAC transcription factors are involved in the regulation of biotic and abiotic stress responses in plants and mitochondrial retrograde regulation (Ng *et al.* 2014). The DEGs identified in this study could be used as candidate genes in association mapping studies of stress-related traits to discover alleles and identify polymorphisms useful in plant breeding. Similar approaches have been successfully used to identify molecular markers for selection in other crops (Yu *et al.* 2013, 2015; Jespersen *et al.* 2017; Rakoczy-Trojanowska *et al.* 2017).

## Supplementary Material

Supplemental material is available online at www.g3journal.org/lookup/suppl/doi:10.1534/g3.117.300321/-/DC1.
