# Supplementary material for: Transcriptome Analyses of Mosaic (MSC) Mitochondrial Mutants of Cucumber in a Highly Inbred Nuclear Background
Source: G3 (Bethesda). 2018 Jan 12;8(3):953–65. doi: 10.1534/g3.117.300321 (PMC5844315; doi:10.1534/g3.117.300321)
Supplement: Supplementary file 1 [file 953FileS1.doc]

**Supplemental Table 1.** Cucumber genes and primer sequences tested as potential references for reverse transcriptase quantitative (RT-qPCR).

| **Abbr.** | **Gene name** | **Primer sequence (5’ - 3’)** | **Function** | **Orgin** |
| --- | --- | --- | --- | --- |
| ATP | ATPase subunit III | Proprietary by manufacturer | subunit III of ATPase | Cucumber geNorm Kit (Primerdesign Ltd) |
| CACS | Clathrin adaptor complex subunit | TGGGAAGATTCTTATGAAGTGC | intracellular protein transport, vesicle mediated transport | Migocka and Papierniak 2010 |
|  |  | CTCGTCAAATTTACACATTGGT |  |  |
| EFα | Elongation factor 1-alpha | ACTTTATCAAGAACATGATTAC | translational elongation | Migocka and Papierniak 2010 |
|  |  | TTCCTTCACAATTTCATCG |  |  |
| F-box | F-box protein/ galactose oxidase/ kelch repeat protein | GGTTCATCTGGTGGTCTT | unknown | Migocka and Papierniak 2010 |
|  |  | CTTTAAACGAACGGTCAGTCC |  |  |
| GRI | Glutamyl-tRNA reductase, isozyme 1 | Proprietary by manufacturer | tetrapyrroles biosynthesis, activation of the glutamine residues into the ribosomal protein biosynthesis, reduction glutamate in plastids | Cucumber geNorm Kit (Primerdesign Ltd) |
| CYCL | Cyclophilin | AACAGATGCTAGCTCCATCGC | protein folding, signal transduction | Kowalczuk *et al.* 2014 |
|  |  | TCACACAGACCCATATTCGCA |  |  |
| PLD | Phospholipase D | Proprietary by manufacturer | phosphatidic acid production (PA), signal transduction | Cucumber geNorm Kit (Primerdesign Ltd) |
| TIP41 | TIP41-like family  protein | CAACAGGTGATATTGGATTATGATTATAC | PP2A phosphatase activator | Migocka and Papierniak 2010 |
|  |  | GCCAGCTCATCCTCATATAAG |  |  |
| TUA | α - tubulin | ACGCTGTTGGTGGTGGTAC | structural constituent of cytoskeleton, protein folding | Wan *et al.* 2010 |
|  |  | GAGAGGGGTAAACAGTGAATC |  |  |
| TUB | α - tubulin | CCTCGACATTGAGCGACCTAAC | structural constituent of cytoskeleton, protein folding | Kowalczuk *et al.* 2014 |
|  |  | CATCCACGTTCAATGCACCA |  |  |
| UBI-ep | Ubiquitin extension protein | CACCAAGCCCAAGAAGATC | protein binding, protein modification | Wan *et al.* 2010 |
|  |  | TAAACCTAATCACCACCAGC |  |  |

**Literature Cited:**

1. Kowalczuk, C., M. Pawełkowicz, K. Chądzyńska, A. Paziewska, R. Wóycicki *et al.*, 2014 Evaluation of reference genes transcription stability in generative whorls of cucumber flower (*Cucumis sativus* L.), pp. 275–286 in *Biotechnology and plant breeding perspectives*, edited by R. K. Behl, and E. Arseniuk, Agrobios International, Jodhpur, India.
2. Migocka M., and A. Papierniak, 2010 Identification of suitable reference genes for studying gene expression in cucumber plants subjected to abiotic stress and growth regulators. Mol. Breed. 28: 343–57.
3. Wan, H., Z. Zhao, C. Qian, Y. Sui, A. A. Malik *et al.*, 2010 Selection of appropriate reference genes for gene expression studies by quantitative real-time polymerase chain reaction in cucumber. Anal. Biochem. 399: 257–261.

**Supplemental Table 2.** Overall comparison of expression profiles for 11 putative reference genes. Results are calculated for all samples for each line MSC3, 12, 16, and wild-type B. Rankings are based on geNorm stability M-values, NormFinder stability values and BestKeeper coefficient of correlation values (R). Standard deviation (SD) values calculated by BestKeeper are also given in the table. Genes *GRI* with the highest average expression stability, and *EFα* with SD>1 was eliminated from further analysis. For the overall final ranking the geometric mean of the weights (GeoMean) assigned by the rankings from all three programs was calculated. Genes selected as references are shown in bold text in Final Ranking column.

| **Rank.**  **(weight)** | **Program** | | | | | | | **Final Ranking** | |
| --- | --- | --- | --- | --- | --- | --- | --- | --- | --- |
|  | **geNorm** | | **NormFinder** | | **BestKeeper** | | |  |  |
|  | Gene | M-value | Gene | Stability value | Gene | R | SD | Gene | GeoMean |
| 1 | UBI-ep | 0.197 | TIP41 | 0.083 | TUA | 0.973 | 0.515 | **TIP41** | **0.251** |
| 2 | TIP41 | 0.197 | F-box | 0.099 | TIP41 | 0.971 | 0.291 | **F-box** | **0.307** |
| 3 | ATP | 0.231 | CACS | 0.143 | PLD | 0.966 | 0.566 | **UBI-ep** | **0.316** |
| 4 | CACS | 0.286 | TUA | 0.157 | F-box | 0.953 | 0.494 | ATP | 0.324 |
| 5 | F-box | 0.307 | ATP | 0.173 | TUB | 0.944 | 0.425 | CACS | 0.335 |
| 6 | TUA | 0.321 | UBI-ep | 0.179 | CACS | 0.923 | 0.459 | TUA | 0.366 |
| 7 | PLD | 0.347 | PLD | 0.223 | UBI-ep | 0.895 | 0.281 | PLD | 0.421 |
| 8 | EFα | 0.499 | TUB | 0.229 | EFα | 0.885 | 1.085 | CYCL | 0.693 |
| 9 | CYCL | 1.722 | CYCL | 0.240 | ATP | 0.852 | 0.261 | TUB | 0.842 |
| 10 | TUB | 2.757 | GRI | 0.489 | CYCL | 0.804 | 0.233 | EFα | - |
| 11 | GRI | 3.754 | EFα | 0.685 | GRI | - | - | GRI | - |

**Supplemental** **Table 3.** Primer sequences used for validation of differentially expressed genes in mitochondrial mutants MSC3, 12, and 16 relative to wild-type cucumber and Real-Time qPCR amplification efficiencies (E).

| **Cucumber unigene** | **Abbr.** | **Primer sequence (5’ - 3’)** | | **Amplicon size in bp** | **%E** |
| --- | --- | --- | --- | --- | --- |
|  |  | **Forward** | **Reverse** |  |  |
| Csa1M476010 | **HIPP26** | CCCGGATGTGAGAAACAAGT | GCACTGCCTTCAGGATCTTC | 133 | 103.6 |
| Csa1M537480 | **1-BBE** | GCCTTGTTTCTTGGAGATTCA | AGAGCACAGATTGAAGCCAAC | 160 | 102.2 |
| Csa1M539350 | **2-BBE** | AAGGAACCCATCCCTAAAGC | CAGCTCTATGCGAGAAAGGA | 148 | 103.9 |
| Csa1M595860 | **CYP-like** | ATGGAGGAGTGCAAGCAGTT | GACCTCCTGCCTAGCCTTTT | 120 | 103.3 |
| Csa1M600240 | **BPM4** | CAAATCCCCGTCGCTTTAT | TTGTGCAAGCACTTTTCCAC | 159 | 104.6 |
| Csa2M360680 | **1-SerpinZX** | ACGTTGTTCTAGCCGTTTGC | ACCATCTGCCCAACAAAAAG | 134 | 104.0 |
| Csa2M360690 | **2-SerpinZX** | ATTCTTCTCCCCGGATCAGT | CTGCTCATGAAAGGGGTCTT | 161 | 101.8 |
| Csa3M020080 | **1-HSP23** | CCTGGTTTCGGAGATGTGTT | GCTCCTACTCCTCGTGATGC | 119 | 99.9 |
| Csa3M020090 | **2-HSP23** | CTTTCTCCGCACCTAAACTCA | GCGGTCCACTTCAACAGAAC | 134 | 102.8 |
| Csa3M167380 | **CaBP1** | GACTTGAATCGGAACGGAGT | CAACCGTCCATCTCCATCTT | 129 | 102.1 |
| Csa3M819830 | **WRNexo** | TTTCACTTTTGTTGGCGTTG | CCGGCAGTCCTAAACTCATC | 138 | 104.4 |
| Csa3M829160 | **SAP12** | AAGGTTCTGGAGAGGCATCA | AACCGGCAGGTTTTACACAC | 131 | 102.2 |
| Csa4M193250 | **NAC87** | CTTGGGATTTGCCTCAGAAG | AAGAACCGACTTCCCCTTGT | 167 | 97.5 |
| Csa4M285790 | **POX53** | GCAGCTCCAAACAATGGAAT | ACAAGAAACAACGCCTGGAC | 129 | 101.5 |
| Csa4M303690 | **GSTu8** | TGGGTTTGTGGACATTGTTG | TGGTTGACAATGGAATGCTG | 147 | 105.1 |
| Csa4M304250 | **GST-like** | GACTGTGATTGCTGGTTGGA | GCTGATCACTCCATTTGATTAGG | 101 | 104.1 |
| Csa4M639960 | **unAT** | TTTCAAGCAGAGGACGACAA | GGGTGCTTCGAATTTGGTAA | 135 | 100.2 |
| Csa5M155520 | **HAP2** | GAGTGGAAGGACGGTTTGAG | CATCATCAGCACGCAATTCT | 120 | 101.8 |
| Csa5M577370 | **NUDX1** | TGTCACCATTTCTATGCGAGCT | ATCCCAGTCATACCAGTCCCA | 100 | 97.5 |
| Csa5M590010 | **Ntn** | CGACGACATGAGTTGGAAAA | ACCAGTTTCATCCCATCCAG | 135 | 103.7 |
| Csa6M006690 | **unPLAC** | TGCTTGCTTGGAAGTGAAGA | AGCCATGGGTGGTAATTGAG | 159 | 103.5 |
| Csa6M042450 | **CRF6** | TGCTCTCCAACTTCGGTTCT | CTCCGGTAAGCTCCTTTCCT | 129 | 104.1 |
| Csa6M094680 | **PPI1** | GCACCATGGAAGAAGGTGAT | TGTCGAACAACTCCAACAGG | 136 | 104.4 |
| Csa6M094690 | **PPI2** | GGAACACCCATTGCTTTGAT | AGCTCATCACCAGCAGGTTT | 133 | 101.6 |
| Csa6M154530 | **PGA55** | TCCCAATGACCCTGAAAGAG | TGCTGCGAACAGAATCTACG | 155 | 99.2 |
| Csa6M504470 | **1-unFtsH** | TGAGGAATCACCCTTTGCAT | TCGTCGATTATCCATTGCTTC | 105 | 101.7 |
| Csa6M504480 | **2-unFtsH** | AGATCTTTAGCCAAGAATGTAATGAT | TGCTTCAACTCAGGGTCCAC | 121 | 104.2 |
| Csa6M517010 | **NDA2** | GAGAGGGAAGGGAAGTATCTTG | CAACTGATGCCATGCTTCC | 155 | 102.4 |
| Csa6M517020 | **NDA1** | TTCTTGCACCGGTGTTGATA | AGTCAAGGGCTCAGATCCAA | 139 | 96.3 |
| Csa6M518170 | **NAC73** | TACCAGCGGGAGTGAAGTTT | CATCATCTCCATCCAACGTG | 132 | 106.9 |
| Csa7M170600 | **RL1** | CGATGTCTTCCCATGGATCT | AGCAGTTTTTCCACCCACAG | 121 | 98.9 |

**Supplemental Table 4.** Alignment statistics for first (rep1) and second (rep2) biological replicates from non-normalized cDNA libraries of the wild-type inbred B and mitochondrial mutants MSC3, 12, and 16 [SRA accession no. SAMN06768506-SAMN06768513] mapped to the reference sequence of cucumber line 9930 v2 (Huang *et al.* 2009).

| **Sample** | | **Alignment Quality Control** | | | | | | **Total mapped reads [%]** |
| --- | --- | --- | --- | --- | --- | --- | --- | --- |
|  |  | **Total reads** | **Clean reads**  **Number [%]** | | **Mapped reads** | **Multi-position match**  **number [%]** | |  |
| Line B | rep1 | 13 716 194 | 10 383 786 | 75.7 | 8 844 858 | 724 116 | 8.2 | 85.2 |
|  | rep2 | 13 581 000 | 10 059 618 | 74.1 | 8 153 120 | 663 876 | 8.1 | 81.0 |
| MSC3 | rep1 | 13 327 324 | 9 940 252 | 74.6 | 8 054 148 | 697 852 | 8.7 | 81.0 |
|  | rep2 | 13 875 806 | 10 413 502 | 75.1 | 8 550 580 | 775 820 | 9.1 | 82.1 |
| MSC12 | rep1 | 12 868 482 | 9 566 202 | 74.3 | 7 876 794 | 630 948 | 8.0 | 82.3 |
|  | rep2 | 12 801 092 | 9 695 206 | 75.7 | 8 115 134 | 720 578 | 8.9 | 83,7 |
| MSC16 | rep1 | 12 895 966 | 9 730 146 | 75.5 | 8 019 992 | 702 622 | 8.8 | 82.4 |
|  | rep2 | 13 213 146 | 9 971 972 | 75.5 | 8 160 986 | 753 372 | 9.2 | 81.8 |

**Supplemental Table 5. Twenty-three DEGs directly or indirectly associated with stress-response pathways based on Gene Ontology classifications combined with analysis of the available scientific reports.**

| **No.** | **Cucumber unigene** | **Abbr.** | **Stress responsivity** | **Reference** |
| --- | --- | --- | --- | --- |
| 1 | Csa1M476010 | **HIPP26** | heavy metals (Cd and Zn), cold, drought and salinity | Abreu-Neto *et al.* 2013 |
| 2 | Csa1M537480 | **1-BBE** | biotic stress and salinity | Daniel *et al.* 2016 |
| 3 | Csa1M539350 | **2-BBE** |
| 4 | Csa2M193320 | **BHLH92** | osmotic stress | Jiang *et al.* 2009 |
| 5 | Csa2M360680 | **1-SerpinZX** | biotic stress - regulation of programmed cell death (PCD) induced by elicitors and drought | Cohen *et al.* 2015,  Koh *et al.* 2016 |
| 6 | Csa2M360690 | **2-SerpinZX** |
| 7 | Csa3M020080 | **1-HSP23** | GO:0006950 - response to stress | Blast2GO v2.7.2 |
| 8 | Csa3M020090 | **2-HSP23** |
| 9 | Csa3M167380 | **CaBP1** | wide spectrum of biotic and abiotic stress | Yáñez *et al.* 2012 |
| 10 | Csa3M819830 | **WRNexo** | replication stress | Knoll and Puchta 2010 |
| 11 | Csa3M829160 | **SAP12** | GO:0006950 - response to stress | Blast2GO v2.7.2 |
| 12 | Csa4M141240 | **1-nsLTP2** | wide spectrum of biotic and abiotic stress | Liu *et al.* 2015 |
| 13 | Csa4M146250 | **1-nsLTP2** |
| 14 | Csa4M193250 | **NAC87** | expression induced by H_2_O_2_ | Inzé *et al.* 2012 |
| 15 | Csa4M285790 | **POX53** | oxidative and environmental stress, including: wounding and pathogen attack. | Tognolli *et al.* 2002  Jin *et al.* 2011 |
| 16 | Csa4M303690 | **GSTu8-like** | oxidative stress, reduction of excess H_2_O_2_ | Edwards *et al.* 2000 |
| 17 | Csa4M304250 | **GST-like** |
| 18 | Csa5M577370 | **NUDIX1** | replication stress | Kraszewska 2008 |
| 19 | Csa6M042450 | **CRF6** | oxidative stress | Zwack *et al.* 2016 |
| 20 | Csa6M504470 | **1-unFtsH** | oxidative stress | Smakowska *et al.* 2014 |
| 21 | Csa6M504480 | **2-unFtsH** |
| 22 | Csa6M517010 | **NDA2** | oxidative stress | **Møller 2001**  Vanlerberghe *et al.* 2009 |
| 23 | Csa6M517020 | **NDA1** |

**Literature Cited:**

1. Abreu-Neto, J. B., A. C. Turchetto-Zolet, L. F. Valter de Oliveira, M. H. Zanettini, M. Margis-Pinheiro, 2013 Heavy metal-associated isoprenylated plant protein (HIPP): characterization of a family of proteins exclusive to plants. FEBS J. 280: 1604–1616.
2. Cohen, M., T. H. Roberts, R. Fluhr, 2015 Serpins in plants, pp. 15-28 in: *The Serpin family: proteins with multiple functions in health and disease*, edited by M. Geiger, F. Wahlmüller, M. Furtmüller, Springer International Publishing.
3. Daniel, B., S. Wallner, B. Steiner, G. Oberdorfer, P. Kumar *et al*., 2016 Structure of a Berberine Bridge Enzyme-Like enzyme with an active site specific to the plant family *Brassicaceae*. PLoS ONE 11: e0156892.
4. Edwards, R., D. P. Dixon, V. Walbot, 2000 Plant glutathione S-transferases: enzymes with multiple functions in sickness and in health. Trends Plant Sci. 5: 193–198.
5. Inzé, A., S. Vanderauwera, F. A. Hoeberichts, M. Vandorpe, T. Van Gaever *et al*., 2012 A subcellular localization compendium of hydrogen peroxide-induced proteins. Plant Cell Environ. 35: 308–320.
6. Jiang, Y., B. Yang, M. K. Deyholos, 2009 Functional characterization of the *Arabidopsis* bHLH92 transcription factor in abiotic stress. Mol. Genet. Genomics 282: 503–519.
7. Jin, J., T. Hewezi, T. J. Baum, 2011 *Arabidopsis* peroxidase AtPRX53 influences cell elongation and susceptibility to *Heterodera schachtii*. Plant Signal Behav. 11: 1778–1786.
8. Kraszewska, E., 2008 The plant Nudix hydrolase family. Acta Biochim. Pol. 55: 663–671.
9. Knoll, A., and H. Puchta, 2011 The role of DNA helicases and their interaction partners in genome stability and meiotic recombination in plants. J. Exp. Bot. 62: 1565–1579.
10. Koh, E., R. Carmieli, A. Mor, R. Fluhr, 2016 Singlet oxygen induced membrane disruption and serpin-protease balance in 21 vacuolar driven cell death in *Arabidopsis thaliana*. Plant Physiol. 171: 1616–1625.
11. Liu, F., X. Zhang, C. Lu, X. Zeng, Y. Li *et al*., 2015 Non-specific lipid transfer proteins in plants: presenting new advances and an integrated functional analysis. J. Exp. Bot. 66: 5663–5681.
12. **Møller, I. M.**, 2001 Plant mitochondria and oxidative stress: electron transport, NADPH turnover, and metabolism of reactive oxygen species. Annu. Rev. Plant Physiol. Plant Mol. Biol. 52**:** 561–591.
13. Smakowska, E., M. Czarna, H. Jańska, 2014 Mitochondrial ATP-dependent proteases in protection against accumulation of carbonylated proteins. Mitochondrion 19: 245–251.
14. Tognolli, M., C. Penel, H. Greppin, P. Simon, 2002 Analysis and expression of the class III peroxidase large gene family in *Arabidopsis thaliana*. Gene 288: 129-138.
15. Vanlerberghe, G. C., M. Cvetkovska, J. Wang, 2009 Is the maintenance of homeostatic mitochondrial signaling during stress a physiological role for alternative oxidase? Physiol. Plant. 137: 392‑406.
16. Yáñez, M., J. Gil-Longo, M. Campos-Toimil, 2012 Calcium Binding Proteins, pp. 461‑482 in: *Calcium Signaling*, edited by S. Islam, Springer Netherlands, The Netherlands.
17. Zwack, P. J., I. De Clercq, T. C. Howton, 2016 Cytokinin Response Factor 6 represses cytokinin-associated genes during oxidative stress. Plant Physiol., 172: 1249–1258.

**Supplemental Table 6.** Real-Time quantitative PCR validation of differentially expressed genes in mitochondrial mutants MSC3, 12 and 16. Mean corrected efficiency Cq (Mean Cq) and normalized expression levels for differentially expressed genes of the MSC mutants relative to wild-type inbred B ± standard error of mean (SEM). Gene names are described in Table 1. Regulation of gene expression are presented as ↑ = up-regulated, ↓ = down-regulated, and const. = no changes. Significance levels (SL) are p < 0.05 (*),  0.01 (**) and  0.001 (***).

| **Gene** | **Sample** | **Mean Cq** | **Normalized expression level** | **Relative normalized expression level** | **SEM** | **Regulation** | **P value** | **SL** |
| --- | --- | --- | --- | --- | --- | --- | --- | --- |
| **DNA REPAIR MECHANISMS** | | | | | | | | |
| **WRNexo**  Csa3M819830 | B | 36.83 | 0.00042 | 1 | 0.28 | control | - | - |
|  | 3 | 34.78 | 0.00165 | 3.96827 | 0.95 | ↑ | 0.047 | * |
|  | 12 | 32.25 | 0.00876 | 21.04125 | 2.37 | ↑ | 0.000 | *** |
|  | 16 | 32.81 | 0.00598 | 14.34962 | 2.11 | ↑ | 0.002 | ** |
| **NUDIX1**  Csa5M577370 | B | 28.89 | 0.10318 | 1 | 0.11 | control | - | - |
|  | 3 | 26.8 | 0.44821 | 4.3438 | 0.49 | ↑ | 0.000 | *** |
|  | 12 | 26.31 | 0.55627 | 5.39114 | 0.34 | ↑ | 0.000 | *** |
|  | 16 | 25.79 | 0.80797 | 7.83047 | 0.95 | ↑ | 0.000 | *** |
| **REGULATION OF TRANSCRIPTION** | | | | | | | | |
| **NAC87**  Csa4M193250 | B | 31.45 | 0.01942 | 1 | 0.17 | control | - | - |
|  | 3 | 29.31 | 0.08448 | 4.34949 | 0.52 | ↑ | 0.000 | *** |
|  | 12 | 28.93 | 0.09873 | 5.08319 | 0.30 | ↑ | 0.000 | *** |
|  | 16 | 28.79 | 0.10668 | 5.49262 | 0.51 | ↑ | 0.000 | *** |
| **CRF6**  Csa6M042450 | B | 34.93 | 0.00182 | 1 | 0.21 | control | - | - |
|  | 3 | 33.61 | 0.00437 | 2.39742 | 0.58 | ↑ | 0.026 | * |
|  | 12 | 32.7 | 0.00752 | 4.12955 | 0.68 | ↑ | 0.000 | *** |
|  | 16 | 32.18 | 0.01063 | 5.83577 | 0.72 | ↑ | 0.000 | *** |
| **NAC73**  Csa6M518170 | B | 34.78 | 0.00198 | 1 | 0.19 | control | - | - |
|  | 3 | 28.79 | 0.1268 | 63.96019 | 7.28 | ↑ | 0.000 | *** |
|  | 12 | 29 | 0.09501 | 47.92607 | 6.30 | ↑ | 0.000 | *** |
|  | 16 | 28.86 | 0.10823 | 54.59054 | 6.94 | ↑ | 0.000 | *** |
| **RL1**  Csa7M170600 | B | 25.75 | 1.31889 | 1 | 0.25 | control | - | - |
|  | 3 | 26.67 | 0.66834 | 0.50675 | 0.12 | ↓ | 0.039 | * |
|  | 12 | 25.34 | 1.44306 | 1.09415 | 0.08 | const. | 0.715 | - |
|  | 16 | 25.69 | 1.1453 | 0.86838 | 0.08 | const. | 0.203 | - |
| **SIGNAL TRANDUCTION MECHANISMS** | | | | | | | | |
| **CaBP1**  Csa3M167380 | B | 30.81 | 0.03845 | 1 | 0.12 | control | - | - |
|  | 3 | 28.18 | 0.22915 | 5.96004 | 0.93 | ↑ | 0.000 | *** |
|  | 12 | 27.95 | 0.24843 | 6.46134 | 0.32 | ↑ | 0.000 | *** |
|  | 16 | 27.77 | 0.2748 | 7.14737 | 0.62 | ↑ | 0.000 | *** |
| **CELLULAR METABOLIC PROCESSES** | | | | | | | | |
| **GST8u-like**  Csa4M303690 | B | 33.64 | 0.00432 | 1 | 0.15 | control | - | - |
|  | 3 | 29.82 | 0.05894 | 13.62836 | 2.01 | ↑ | 0.000 | *** |
|  | 12 | 29.51 | 0.06237 | 14.42217 | 1.31 | ↑ | 0.000 | *** |
|  | 16 | 29.56 | 0.06096 | 14.09505 | 1.89 | ↑ | 0.000 | *** |
| **GST-like**  Csa4M304250 | B | 35.03 | 0.00181 | 1 | 0.29 | control | - | - |
|  | 3 | 28.85 | 0.12665 | 69.83972 | 12.80 | ↑ | 0.000 | *** |
|  | 12 | 28.26 | 0.1655 | 91.26307 | 13.41 | ↑ | 0.000 | *** |
|  | 16 | 28.1 | 0.18701 | 103.1199 | 11.67 | ↑ | 0.000 | *** |
| **unAT**  Csa4M639960 | B | 34.74 | 0.00207 | 1 | 0.33 | control | - | - |
|  | 3 | 29.5 | 0.0773 | 37.39425 | 10.89 | ↑ | 0.007 | ** |
|  | 12 | 29.42 | 0.07443 | 36.0021 | 3.80 | ↑ | 0.000 | *** |
|  | 16 | 29.09 | 0.09092 | 43.97877 | 9.92 | ↑ | 0.000 | *** |
| **1-BBE**  Csa1M537480 | B | 37.3 | 0.00034 | 1 | 0.41 | control | - | - |
|  | 3 | 35.21 | 0.00137 | 4.07584 | 1.04 | ↑ | 0.002 | ** |
|  | 12 | 34.92 | 0.00146 | 4.34813 | 0.72 | ↑ | 0.005 | ** |
|  | 16 | 34.93 | 0.00146 | 4.33711 | 1.06 | ↑ | 0.019 | * |
| **2-BBE**  Csa1M539350 | B | 36.88 | 0.00046 | 1 | 0.31 | control | - | - |
|  | 3 | 29.58 | 0.06879 | 149.2879 | 35.05 | ↑ | 0.000 | *** |
|  | 12 | 29.2 | 0.07802 | 169.3265 | 24.16 | ↑ | 0.000 | *** |
|  | 16 | 29.03 | 0.09103 | 197.5523 | 17.68 | ↑ | 0.000 | *** |
| **CYP-like**  Csa1M595860 | B | 35.14 | 0.00163 | 1 | 0.24 | control | - | - |
|  | 3 | 30.18 | 0.04993 | 30.61031 | 8.67 | ↑ | 0.003 | ** |
|  | 12 | 29.33 | 0.08094 | 49.61797 | 3.45 | ↑ | 0.000 | *** |
|  | 16 | 29.31 | 0.08138 | 49.88746 | 6.15 | ↑ | 0.000 | *** |
| **POX53**  Csa4M285790 | B | 36.77 | 0.00045 | 1 | 0.26 | control | - | - |
|  | 3 | 36.08 | 0.00071 | 1.5586 | 0.62 | const. | 0.330 | - |
|  | 12 | 34.77 | 0.00152 | 3.35905 | 0.40 | ↑ | 0.009 | ** |
|  | 16 | 35.31 | 0.00106 | 2.33504 | 0.73 | const. | 0.175 | - |
| **PPI1**  Csa6M094680 | B | 37.64 | 0.00029 | 1 | 0.56 | control | - | - |
|  | 3 | 33.74 | 0.00422 | 14.57807 | 4.60 | ↑ | 0.030 | * |
|  | 12 | 34.1 | 0.00284 | 9.82092 | 1.07 | ↑ | 0.000 | *** |
|  | 16 | 33.61 | 0.0041 | 14.15754 | 1.79 | ↑ | 0.000 | *** |
| **PPI2**  Csa6M094690 | B | 32.89 | 0.0082 | 1 | 0.12 | control | - | - |
|  | 3 | 29.38 | 0.09012 | 10.99573 | 2.04 | ↑ | 0.000 | *** |
|  | 12 | 29.76 | 0.06274 | 7.65464 | 0.58 | ↑ | 0.000 | *** |
|  | 16 | 29.67 | 0.06653 | 8.11774 | 0.51 | ↑ | 0.000 | *** |
| **NDA2**  Csa6M517010 | B | 30.54 | 0.04104 | 1 | 0.11 | control | - | - |
|  | 3 | 29.26 | 0.09528 | 2.32186 | 0.27 | ↑ | 0.000 | *** |
|  | 12 | 28.8 | 0.11993 | 2.9224 | 0.14 | ↑ | 0.000 | *** |
|  | 16 | 28.66 | 0.12965 | 3.15926 | 0.12 | ↑ | 0.000 | *** |
| **NDA1**  Csa6M517020 | B | 32.13 | 0.01429 | 1 | 0.13 | control | - | - |
|  | 3 | 28.48 | 0.17751 | 12.42518 | 1.70 | ↑ | 0.000 | *** |
|  | 12 | 27.78 | 0.2477 | 17.33874 | 1.45 | ↑ | 0.000 | *** |
|  | 16 | 27.62 | 0.28455 | 19.91754 | 1.72 | ↑ | 0.000 | *** |
| **RESPONSE TO STIMULUS** | | | | | | | | |
| **1-HSP23**  Csa3M020080 | B | 28.35 | 0.18002 | 1 | 0.12 | control | - | - |
|  | 3 | 24.59 | 2.56298 | 14.23698 | 2.32 | ↑ | 0.000 | *** |
|  | 12 | 23.96 | 3.37707 | 18.75918 | 1.85 | ↑ | 0.000 | *** |
|  | 16 | 24.19 | 2.81662 | 15.64593 | 2.60 | ↑ | 0.000 | *** |
| **2-HSP23**  Csa3M020090 | B | 32.69 | 0.00736 | 1 | 0.23 | control | - | - |
|  | 3 | 30.95 | 0.02295 | 3.11662 | 1.11 | ↑ | 0.017 | * |
|  | 12 | 30.07 | 0.03739 | 5.07781 | 0.79 | ↑ | 0.000 | *** |
|  | 16 | 29.85 | 0.04342 | 5.89568 | 1.28 | ↑ | 0.003 | ** |
| **SAP12**  Csa3M829160 | B | 31.37 | 0.02524 | 1 | 0.09 | control | - | - |
|  | 3 | 28.86 | 0.13766 | 5.45417 | 0.98 | ↑ | 0.000 | *** |
|  | 12 | 28.57 | 0.15523 | 6.15037 | 0.25 | ↑ | 0.000 | *** |
|  | 16 | 28.61 | 0.14875 | 5.89345 | 0.66 | ↑ | 0.000 | *** |
| **REGULATION OF PROTEOLYTIC PROCESSES** | | | | | | | | |
| **1-SerpinZX**  Csa2M360680 | B | 37.41 | 0.00029 | 1 | 0.54 | control | - | - |
|  | 3 | 31.76 | 0.01402 | 48.1636 | 7.28 | ↑ | 0.000 | *** |
|  | 12 | 30.67 | 0.02613 | 89.7419 | 7.69 | ↑ | 0.000 | *** |
|  | 16 | 30.32 | 0.03303 | 113.4317 | 11.55 | ↑ | 0.000 | *** |
| **2-SerpinZX**  Csa2M360690 | B | 35.17 | 0.00161 | 1 | 0.22 | control | - | - |
|  | 3 | 28.43 | 0.16482 | 102.5249 | 24.69 | ↑ | 0.003 | ** |
|  | 12 | 28.01 | 0.1888 | 117.4423 | 10.12 | ↑ | 0.000 | *** |
|  | 16 | 28.1 | 0.18055 | 112.3087 | 14.51 | ↑ | 0.000 | *** |
| **Ntn**  Csa5M590010 | B | 33.27 | 0.00679 | 1 | 0.17 | control | - | - |
|  | 3 | 30.78 | 0.0361 | 5.31708 | 1.06 | ↑ | 0.000 | *** |
|  | 12 | 30.62 | 0.03643 | 5.36686 | 0.24 | ↑ | 0.000 | *** |
|  | 16 | 30.5 | 0.03957 | 5.82886 | 0.72 | ↑ | 0.000 | *** |
| **1-unFtsH**  Csa6M504470 | B | 36.22 | 0.00073 | 1 | 0.20 | control | - | - |
|  | 3 | 31.98 | 0.01304 | 17.95729 | 2.51 | ↑ | 0.000 | *** |
|  | 12 | 31.49 | 0.01669 | 22.97482 | 1.72 | ↑ | 0.000 | *** |
|  | 16 | 31.44 | 0.0171 | 23.53542 | 1.96 | ↑ | 0.000 | *** |
| **2-unFtsH**  Csa6M504480 | B | 37.54 | 0.00034 | 1 | 0.21 | control | - | - |
|  | 3 | 35.46 | 0.00136 | 4.01048 | 1.00 | ↑ | 0.034 | * |
|  | 12 | 34.79 | 0.00197 | 5.79909 | 1.15 | ↑ | 0.034 | * |
|  | 16 | 35.38 | 0.00131 | 3.86132 | 0.59 | ↑ | 0.010 | * |
| **TRANSPORT OF METAL IONS/LIPIDS** | | | | | | | | |
| **HIPP26**  Csa1M476010 | B | 33.58 | 0.00437 | 1 | 0.26 | control | - | - |
|  | 3 | 31.73 | 0.01528 | 3.49407 | 0.61 | ↑ | 0.000 | *** |
|  | 12 | 31.19 | 0.01905 | 4.35616 | 0.21 | ↑ | 0.000 | *** |
|  | 16 | 31.83 | 0.01233 | 2.81937 | 0.26 | ↑ | 0.000 | *** |
| **OTHER PROTEINS** | | | | | | | | |
| **BPM4**  Csa1M600240 | B | 36.3 | 0.00077 | 1 | 0.32 | control | - | - |
|  | 3 | 32.3 | 0.01204 | 15.69433 | 3.32 | ↑ | 0.000 | *** |
|  | 12 | 31.51 | 0.01846 | 24.06395 | 3.33 | ↑ | 0.000 | *** |
|  | 16 | 31.74 | 0.01566 | 20.4225 | 2.98 | ↑ | 0.000 | *** |
| **HAP2**  Csa5M155520 | B | 33.07 | 0.00642 | 1 | 0.29 | control | - | - |
|  | 3 | 31.08 | 0.02481 | 3.86469 | 0.86 | ↑ | 0.015 | * |
|  | 12 | 30.78 | 0.02764 | 4.30476 | 0.38 | ↑ | 0.001 | ** |
|  | 16 | 30.81 | 0.02649 | 4.12578 | 0.54 | ↑ | 0.002 | ** |
| **unPLAC8**  Csa6M006690 | B | 35.85 | 0.00105 | 1 | 0.21 | control | - | - |
|  | 3 | 32.29 | 0.01272 | 12.15034 | 3.24 | ↑ | 0.004 | ** |
|  | 12 | 31.48 | 0.02044 | 19.52802 | 2.21 | ↑ | 0.000 | *** |
|  | 16 | 31.09 | 0.02502 | 23.90627 | 2.51 | ↑ | 0.000 | *** |
| **PGA55**  Csa6M154530 | B | 30.4 | 0.03666 | 1 | 0.14 | control | - | - |
|  | 3 | 27.42 | 0.2874 | 7.83964 | 1.23 | ↑ | 0.000 | *** |
|  | 12 | 27.5 | 0.24256 | 6.61663 | 0.56 | ↑ | 0.000 | *** |
|  | 16 | 27.26 | 0.29756 | 8.11678 | 0.85 | ↑ | 0.000 | *** |
